# Management of epilepsy in brain tumor patients

**DOI:** 10.1097/CCO.0000000000000876

**Published:** 2022-07-16

**Authors:** Pim B. van der Meer, Martin J.B. Taphoorn, Johan A.F. Koekkoek

**Affiliations:** a Department of Neurology, Leiden University Medical Center, Leiden; b Department of Neurology, Haaglanden Medical Center, The Hague, The Netherlands

**Keywords:** antiepileptic drug, brain tumor, epilepsy, glioma, levetiracetam, seizure, valproic acid

## Abstract

A concise review of recent findings in brain tumor-related epilepsy (BTRE), with focus on the effect of antitumor treatment on seizure control and the management of antiepileptic drugs (AEDs).

**Recent findings:**

Isocitrate dehydrogenase mutation and its active metabolite d-2-hydroxyglutarate seem important contributing factors to epileptogenesis in BTRE. A beneficial effect of antitumor treatment (i.e. surgery, radiotherapy, and chemotherapy) on seizure control has mainly been demonstrated in low-grade glioma. AED prophylaxis in seizure-naïve BTRE patients is not recommended, but AED treatment should be initiated after a first seizure has occurred. Comparative efficacy randomized controlled trials (RCTs) are currently lacking, but second-generation AED levetiracetam seems the preferred choice in BTRE. Levetiracetam lacks significant drug-drug interactions, has shown favorable efficacy compared to valproic acid in BTRE, generally causes no hematological or neurocognitive functioning adverse effects, but caution should be exercised with regard to psychiatric adverse effects. Potential add-on AEDs in case of uncontrolled seizures include lacosamide, perampanel, and valproic acid. Ultimately, in the end-of-life phase when oral intake of medication is hampered, benzodiazepines via nonoral administration routes are potential alternatives.

**Summary:**

Management of seizures in BTRE is complex and with currently available evidence levetiracetam seems the preferred choice. Comparative efficacy RCTs in BTRE are warranted.

## INTRODUCTION

Epileptic seizures occur frequently in brain tumor patients and often represent the first clinical sign of a brain tumor, although the incidence varies greatly between different tumor types. An epileptic seizure is conceptually defined as: ‘a transient occurrence of signs and/or symptoms due to abnormal excessive or synchronous neuronal activity in the brain’. Epilepsy is a brain disorder and is characterized by an abnormally increased predisposition to epileptic seizures, and was usually applied in clinical practice as having two unprovoked seizures, >24 h apart [1]. In 2014, the International League Against Epilepsy (ILAE) proposed the following practical clinical definition of epilepsy: ‘≥2 unprovoked (or reflex) seizures occurring >24 h apart; one unprovoked (or reflex) seizure and a probability of further seizures similar to the general recurrence risk (≥60%) after two unprovoked seizures, occurring over the next 10 years; or diagnosis of an epilepsy syndrome [2]’. In case of brain tumors, the recurrence risk is not precisely known, meaning in clinical practice that the diagnosis of epilepsy is usually made after one seizure. Epileptic seizures, especially when uncontrolled, have a negative effect on social and economic participation, morbidity, health-related quality of life, and neurocognitive functioning in brain tumor patients. Therefore, achieving enduring seizure control is one of the main treatment goals in brain tumor patients [3]. In this review, we discuss the epidemiology and epileptogenesis, the effect of antitumor treatment on seizures, and efficacy of AEDs with a focus on adult patients with glioma, meningioma, or brain metastases. 

**Box 1:**
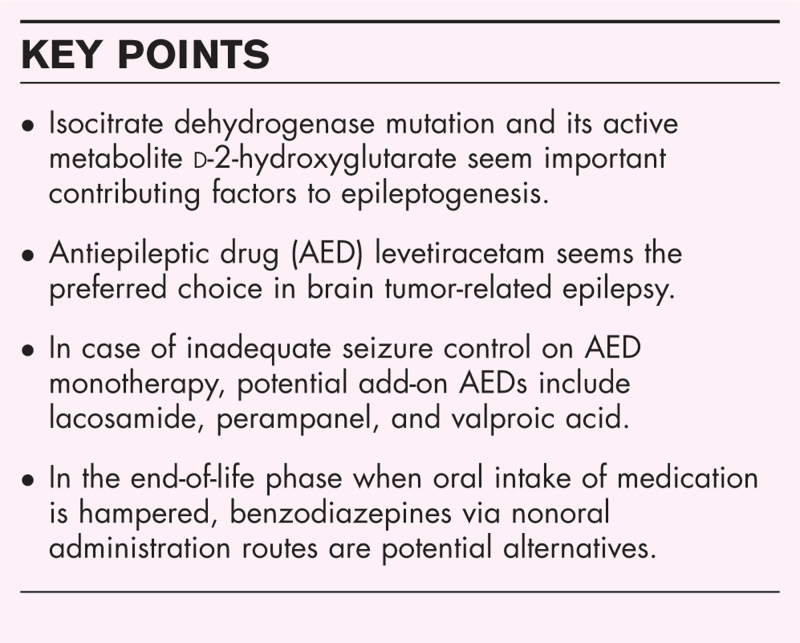
no caption available

## EPIDEMIOLOGY AND EPILEPTOGENESIS

The incidence of seizures is generally higher in patients with low-grade compared to high-grade brain tumors. Up to 100% of grade 1 dysembryoplastic neuroepithelial tumor experience preoperative seizures [4], while this is ∼75% for isocitrate dehydrogenase (IDH-)1/2 mutant grade 2 glioma (either astrocytoma or oligodendroglioma) [5], ∼30% for meningioma [6], ∼25% for glioblastoma IDH-wildtype [5], and ∼20% for patients with brain metastases [7]. Several important clinically relevant factors contributing to epileptogenesis (i.e. the development and extension of brain tissue capable of generating spontaneous recurrent seizures) in brain tumors have been identified, including IDH-mutation and its active metabolite d-2-hydroxyglutarate [8,9], and BRAF V600E mutation (especially in gangliogliomas) [10^▪^,^11^^▪▪^].

## EFFECT OF ANTITUMOR TREATMENT ON SEIZURE CONTROL

### Surgery

Surgical resection of the tumor is the main antitumor treatment strategy in the majority of patients and has been associated with improved overall survival. However, achieving seizure freedom through surgical resection is also a critical objective. Gross total resection is associated with a substantially better seizure outcome in glioma patients compared to nongross total resection [12], which also applies to brain metastases [13], and meningioma [6]. In glioblastoma, supra total surgical resection extends beyond the contrast-enhancing portion of the tumor and has been associated with improved overall survival and seizure control compared to gross total resection [14,15^▪^]. Use of intraoperative electrocorticography significantly improved seizure freedom rates in glioma patients [16]. Previously considered inaccessible tumors in the insular region can usually be successfully accessed and resected with improved imaging and intra-operative monitoring techniques and lead to comparable seizure freedom rates as cortically located tumors [17]. Seizure recurrence after initial postoperative seizure control is associated with tumor progression [6,7,18], but by surgical re-resection patients can regain seizure control [19].

### Radiotherapy

Radiotherapy is often an integral part of the treatment regimen in brain tumor patients aiming to improve local tumor control, preserve and/or improve patient's functioning, and increase overall survival. A beneficial effect of radiotherapy on seizure outcome in brain metastases has not yet been established [13,20], while it has been demonstrated in low-grade glioma patients with seizure freedom rates ranging from 20% after focal radiotherapy to 80% at 6 months after brachytherapy [21]. However, data on the impact of radiotherapy on seizures in brain tumors, especially nonglial tumors, is scarce and therefore no new important findings have been reported in the past years.

### Chemotherapy

Radiotherapy is combined with chemotherapy as part of standard care in the majority of glioma patients. Most commonly administered chemotherapy agents in glioma patients are temozolomide, PCV (i.e. procarbazine, CCNU [lomustine], and vincristine), and lomustine monotherapy. Besides a beneficial effect on survival, chemotherapy has been associated with an improved seizure outcome with seizure freedom rates ranging from 13% to 60% (administered PCV) and 13% to 50% (administered temozolomide) in low-grade glioma patients [21]. However, in elderly glioblastoma patients the beneficial effect of temozolomide on seizure outcome seems minimal [22].

## ANTIEPILEPTIC DRUGS

Recently, the third AED generation has begun with the introduction of a large number of new agents, including lacosamide, brivaracetam, and perampanel. The updated ILAE evidence review from 2013 established as level A class evidence: four AEDs (i.e. carbamazepine, levetiracetam, phenytoin, and zonisamide) for adults with focal onset seizures, two AEDs (i.e. gabapentine and lamotrigine) for elderly with focal onset seizures, and no AEDs for adults with generalized onset tonic–clonic seizures [23].

### When should antiepileptic drugs be initiated?

According to the updated Society for Neuro-Oncology (SNO) and European Association of Neuro-Oncology (EANO) practice guideline on AED prophylaxis, clinicians should not prescribe AEDs to reduce the risk of seizures in seizure-naïve newly diagnosed brain tumor patients (level A class evidence). There is insufficient evidence to recommend prescribing AEDs in seizure-naïve brain tumor patients in the peri- or postoperative period to reduce the risk of seizures (level C class evidence) [24^▪▪^]. Still, this topic is highly debated, mainly due to the fact that current available evidence for the use of primary seizure prophylaxis is minimal and flawed [25]. The prescription patterns of primary AED prophylaxis vary widely between physicians, ranging from 29% among EANO members to 78% among members of the American Association of Neurological Surgeons and the Congress of Neurological Surgeons [26,27]. Glioma patients who experience a first seizure usually necessitate AED treatment due to the high risk of a recurrent seizure, which is common practice among a vast majority (86%) of European physicians [26].

### Comparative efficacy

In a recently published systematic review on the efficacy of AEDs in glioma patients with epilepsy, levetiracetam, phenytoin, and pregabalin, had the highest efficacy as monotherapeutic agents, with levetiracetam showing lower treatment failure compared to the other agents. However, among 66 included studies only two small randomized controlled trials were found, reflecting the relatively low quality of the evidence [28^▪^]. In a retrospective observational cohort study first-line monotherapy levetiracetam was compared with valproic acid in *n* = 1435 glioma patients and found that levetiracetam had favorable efficacy, but a similar level of toxicity [29^▪▪^]. Among physicians treating patients with brain tumors, levetiracetam is most frequently prescribed and considered first choice in reducing seizure frequency, for both mainly focal and bilateral tonic-clonic seizures [26]. This is in contrast with the recently published results of the SANAD II trial in patients with non-BTRE epilepsy. First-line monotherapy levetiracetam showed to be inferior, with regard to effectiveness and cost-effectiveness, compared to lamotrigine and valproic acid in newly diagnosed focal and generalized or unclassifiable non-BTRE epilepsy, respectively [30^▪▪^,^31^^▪▪^]. However, results from non-BTRE studies are not necessarily applicable to BTRE patients given the uniqueness of this population with increased risk for drug–drug interactions and potentially more frequently occurring adverse effects [26,32,33].

Approximately one third of glioma patients continue to have seizures, despite monotherapy AED treatment and (adequate) dose-escalation, and need an add-on AED [29^▪▪^]. Equivalent first choice AEDs to LEV in BTRE according to international physicians treating patients with brain tumors are lacosamide, lamotrigine, and valproic acid [26]. Instead of prescribing two subsequential AED monotherapies before combining AEDs, in BTRE there is a natural tendency towards AED duotherapy as second-line treatment [26,29^▪▪^,34^▪^,^35^]. Ruda *et al.*[36] conducted a prospective observational study in *n* = 93 brain tumor patients with epilepsy evaluating add-on lacosamide, which showed good efficacy and was generally well tolerated. (Add-on) lacosamide showed similar effectiveness compared to (add-on) lamotrigine in *n* = 139 glioma patients [37^▪^]. The duotherapy combination levetiracetam with valproic acid (*n* = 236) showed better efficacy compared to other duotherapy combinations with either levetiracetam or valproic acid (*n* = 119), while level of toxicity was similar [34^▪^]. Brivaracetam and perampanel are both relatively new AEDs and have both shown to be well tolerated and effective in reducing seizure frequency in BTRE patients as add-on, but sample sizes were small [38,39]. Taking rational polytherapy into account, it is recommended to combine AEDs with different mechanisms of action, for example levetiracetam together with either lacosamide, perampanel, or valproic acid. Well conducted studies evaluating the efficacy of AEDs in glioma, but especially in patients with meningioma or brain metastases patients and epilepsy, are currently scarce and comprise an area of research that deserves more attention.

### Drug resistant epilepsy

The ILAE defined drug resistant epilepsy as follows: A failure of adequate trials of two tolerated, appropriately chosen and used antiepileptic drug schedules (whether as monotherapies or in combination) to achieve sustained seizure freedom [40]. Drug resistant epilepsy occurs in about 15% of glioblastoma and about 40% of grade 2 glioma patients [35,41]. IDH-mutation has not been significantly associated with drug-resistant epilepsy. The 4-year cumulative incidence of drug resistant epilepsy was 18% in IDH-mutated versus 11% in IDH-wildtype glioma patients (*P* = 0.26), although these results needs to be interpreted with caution due to a lack of power [42]. In case drug resistant epilepsy occurs, patients should be reviewed for potentially inadequate adherence of AED treatment. In case of adequate medication adherence, an AED might be substituted or added to optimize AED treatment and reduce seizure frequency. In addition, tumor (re)resection or (re)irradiation should be considered to reduce symptom burden in patients with refractory seizures that can be attributed to a single tumor location.

### Adverse effects

Adverse effects are a major cause of AED treatment failure, and may hinder attaining fully effective dosages and medication adherence. Second-generation AEDs have less enzyme-inducing or inhibiting properties and are thought to have improved tolerability compared to first-generation AEDs in certain comparative studies [3]. However, this was not supported by a recent longitudinal cohort study of 30 years including *n* = 1795 newly diagnosed epilepsy patients. The intolerable adverse effect rate was similar between first- and second-generation AEDs [43]. In glioma patients the intolerable adverse effect rate was similar as well between first-generation AED valproic acid and second-generation AED levetiracetam [29^▪▪^]. The most important adverse effects in BTRE patients are of neurological, psychiatric, or hematological origin [3,29^▪▪^]. Common adverse effects of AEDs include dizziness, headache, nausea, and somnolence [3]. First-generation AEDs, such as carbamazepine, phenytoin, and valproic acid are well known to cause drug–drug interactions (e.g. accelerate metabolism of dexamethasone) and have been associated with neurocognitive impairment in (non)BTRE patients [3,44]. Valproic acid is associated with coagulopathy, particularly trombocytopenia [3,45], which may worsen in combination with temozolomide chemotherapy, but generally causes no psychiatric adverse effects [29^▪▪^]. The second-generation AED levetiracetam lacks significant drug-drug interactions and has been associated with improved neurocognitive functioning [46], generally causes no hematological adverse effects [29^▪▪^], but is known for its psychiatric adverse effects (e.g. depression, agitation or psychosis) [29^▪▪^]. It is of paramount importance neuro-oncology professionals take into account the medical history of a patient and are aware of the differences between the AEDs in adverse effects and drug–drug interactions when prescribing AEDs.

### Antiepileptic drug withdrawal

Taking into account the potential adverse effects of AEDs and the efficacy of antitumor treatment in reducing seizure frequency, withdrawal of AEDs after an interval of seizure freedom might be considered. A prospective study in glioma patients showed that 26% (12/46) of patients, who were ≥1 year seizure free from the date of last antitumor treatment, had a recurrent seizure after AED withdrawal compared to 8% (2/25) of patients continuing AED treatment (median follow-up ∼2 years) [47]. In a retrospective AED withdrawal study after tumor resection, 19% (3/16) of BTRE patients had a recurrent seizure (median follow-up ∼3 years) [48]. AED withdrawal could be considered in brain tumor patients, but optimal timing is currently unknown and potential benefits need to be weighted carefully against the potential risk of seizure recurrence, preferably in a shared-decision making process [49].

### Effect of antiepileptic drugs on survival

Pallud *et al.*[50^▪^] reported an improved overall survival in glioblastoma, IDH-wildtype patients prescribed levetiracetam. No survival benefit was found for levetiracetam in a meta-analysis by Chen *et al.*[51^▪^] but levetiracetam may have a more beneficial effect in unmethylated 0(6)-methylguanine-DNA methyltransferase (MGMT) promotor glioblastoma patients. Happold *et al.*[52] performed a pooled analysis of four randomized controlled trials in *n* = 1869 glioblastoma patients and did not find improvement of progression-free or overall survival in patients taking either valproic acid or levetiracetam. Neither was a survival benefit found for AEDs in glioblastoma patients (*n* = 1263) in a Norwegian national registries study nor in glioma patients (*n* = 1435) in a large Dutch propensity score matched cohort [29^▪▪^,^53^].

### End-of-life phase

The end-of-life phase, when symptom burden increases in patients with a malignant brain tumor and antitumor treatment is no longer effective, generally comprises the last 3 months of life. Among the most prevalent symptoms during this period were somnolence, motor deficit, cognitive disturbances, and seizures. The vast majority of glioma patients with epilepsy were prescribed AEDs during the end-of-life phase, but in the last week before death AEDs were withdrawn in ∼20% of patients mainly due to somnolence or dysphagia interfering with the intake of oral medication [54]. Nonoral AEDs that can easily be administered in an out-of-hospital setting, include midazolam (intranasal, as emergency treatment) and clonazepam (buccal, as prophylactic treatment), which seemed to provide comfort among patients and their informal caregivers in the home setting [55,56].

## CONCLUSION AND FUTURE PERSPECTIVES

Both AEDs and antitumor treatment can contribute in achieving seizure control in BTRE. A large variety of factors need to be taken into account when selecting the most appropriate AED for the individual patient, including antitumor treatment, drug-drug interactions, neurological symptoms hampering oral drug intake and a sometimes limited prognosis of the patient. The efficacy of AEDs seems to differ between non-BTRE and BTRE patients and results can therefore not be directly translated from one population to the other. The incidence of seizures varies between the different brain tumor types, but IDH-mutation and its active metabolite d-2-hydroxyglutarate seem to play an important role in epileptogenesis. Trials evaluating the comparative efficacy in BTRE have been lacking in the past decades. Therefore, the results of the currently ongoing STING (first-line levetiracetam versus valproic acid in glioma patients with epilepsy, ClinicalTrials.gov Identifier: NCT030480) and SPRING (prophylactic levetiracetam versus no prophylactic AED in seizure-naïve glioma patients) trial are much awaited and may help guide clinicians in their clinical decision making [57].

## Acknowledgements

*Prior presentations: None.*

### Financial support and sponsorship

*This research received no specific grant from any funding agency in the public, commercial, or not-for-profit sectors.*

### Conflicts of interest

*There are no conflicts of interest.*

## REFERENCES AND RECOMMENDED READING

Papers of particular interest, published within the annual period of review, have been highlighted as:

▪ of special interest
▪▪ of outstanding interest
